# Targeting T cell metabolism in the tumor microenvironment: an anti-cancer therapeutic strategy

**DOI:** 10.1186/s13046-019-1409-3

**Published:** 2019-09-13

**Authors:** Zhongping Yin, Ling Bai, Wei Li, Tanlun Zeng, Huimin Tian, Jiuwei Cui

**Affiliations:** Cancer Center, The First Hospital of Jilin University, Changchun, 130021 Jilin China

**Keywords:** T cell, Immunotherapy, Cancer, Metabolic reprogramming

## Abstract

T cells play important roles in anti-tumor immunity. Emerging evidence has revealed that distinct metabolic changes impact the activation and differentiation of T cells. Tailoring immune responses by manipulating cellular metabolic pathways and the identification of new targets may provide new options for cancer immunotherapy. In this review, we focus on recent advances in the metabolic reprogramming of different subtypes of T cells and T cell functions. We summarize how metabolic pathways accurately regulate T cell development, differentiation, and function in the tumor microenvironment. Because of the similar metabolism in activated T cells and tumor cells, we also describe the effect of the tumor microenvironment on T cell metabolism reprogramming, which may provide strategies for maximal anti-cancer effects and enhancing the immunity of T cells. Thus, studies of T lymphocyte metabolism can not only facilitate the basic research of immune metabolism, but also provide potential targets for drug development and new strategies for clinical treatment of cancer.

## Background

T cells are divided into many subtypes and kill tumors directly or indirectly by synthesizing various biological molecules. Naïve T cells undergo metabolic reprogramming during proliferation, differentiation, and execution of effector functions. In recent years, studies of tumor and immune cell metabolism have shown that unlike resting cells, which mainly function in oxidative phosphorylation (OXPHOS), activated T cells mainly rely on aerobic glycolysis to obtain energy. Additionally, activated T cells can increase the decomposition of glutamine and reduce fatty acid oxidation (FAO) to meet the requirement of energy, cell growth, proliferation, differentiation, and cytokine secretion [1]. Therefore, different types of T cells are metabolically reprogrammed to perform their function.

Cancer cells also undergo metabolic reprogramming by upregulating glycolysis, glutamine decomposition, and lipid metabolism. These metabolic programs provide essential metabolites and energy for malignant proliferation, invasion, metastasis, and adaptation to adverse living conditions [2]. Additionally, cancer cells regulate the differentiation of immune cells in the tumor microenvironment through their metabolites to indirectly promote cancer growth [3]. For example, tumor cell reprogramming inhibits effector T cells (Teffs) infiltration or induces apoptosis, promotes regulatory T cell (Tregs) differentiation, and exerts immunosuppressive functions by accumulating lactic acid, releasing carbon dioxide, etc. [4] Thus, understanding the regulation of tumor-induced metabolic stress on T cells are helpful to improve anti-cancer metabolic immunotherapy.

In this review, we summarize the latest advances in T cell metabolism and attempt to provide new ideas for anti-cancer therapy by targeting T cell metabolism. We also discuss targeted therapeutic measures for T cell metabolism through related pathways to further enhance the anti-cancer effect.

## Metabolic characteristics of T cell subtypes (Figure 1)

Metabolism can be divided into two complex pathways: catabolic processes and anabolic processes. Catabolic processes are critical for cellular proliferation and functions, while anabolic processes are important for cellular growth. Unlike cancer cells, T cell metabolic reprogramming is initiated by T cell receptor (TCR) recognition of antigens in the presence of costimulatory molecules. Transformation of this energy pattern contributes to the functions of rapidly proliferating T cells by providing the necessary raw materials and energy [5]. Besides, the integrity of mitochondria is also crucial for T cell function [6]. Thus, to better regulate immunity against the cancer microenvironment, an increasing number of studies have focused on the molecular mechanisms dictating metabolic reprogramming in different subtypes of T cells.

**Fig. 1:**
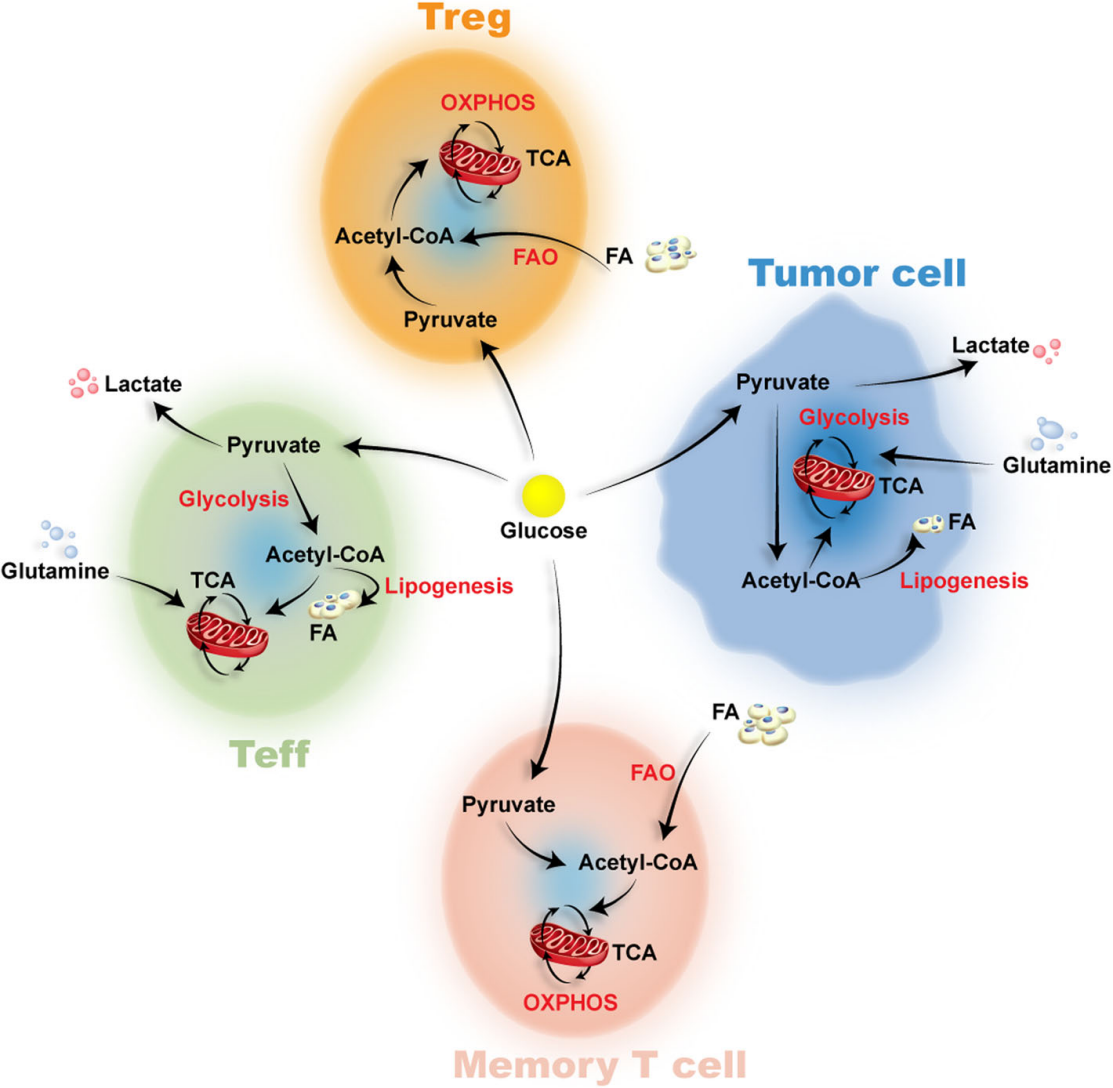
Metabolic reprogramming of T cell subsets in tumor microenvironment. Tumor mainly use glycolysis to produce energy. Glucose competition, amino acid competition, and lactic acid secretion in the tumor microenvironment influence the subsets of T cells. Tregs mainly produce energy by oxidative phosphorylation (OXPHOS) and fatty acid oxidation (FAO). Similar to Treg, memory T cells maintain basic functions by increasing FAO. However, activated Teffs primarily depend on glycolysis and fatty acid synthesis

### Glucose metabolism

T cells undergo metabolic reprogramming during activation, resulting in distinct functional fates [7]. Naïve T cells can generate ATP via OXPHOS. Because glycolysis can produce ATP faster and provide more nutrients for T cell activation than OXPHOS, T cell glucose metabolism occurs through glycolysis as observed in tumors to support their rapid growth and differentiation upon activation by the TCR and costimulatory signals [5]. During this process, naïve T cells shift to Teffs, which can effectively kill tumor cells. In contrast, Tregs and memory CD8^+^ T cells rely on OXOPHOS for survival [8, 9]. This metabolic pattern on memory T cells consumes large amounts of oxygen, which provides an energy basis for the rapid activation and effector function of the body after re-stimulation.

Because T cells at different stages have distinct demands for biological energy and biosynthesis, different signaling pathways are involved in the corresponding metabolic processes [10, 11]. When T cells are activated, phosphoinositide 3-kinase (PI3K)/Akt signaling pathway activation can elevate mTOR signaling, increasing the expression of nutrient transporters (e.g., GLUT-1 or SLC1a5) and further promoting the glycolysis to improve the utilization rate of glucose [12]. Studies have shown that PI3K is related to p85 and p110 in glucose translocation. Akt is involved in regulating glucose metabolism through Forkhead box O1 (FOXO1) and glycogen synthase kinase-3. Additionally, AMP-activated protein kinase inhibited T cells activity by inhibiting mTORC1 activation. A recent study showed that the 6-phosphofructo-2-kinase/fructose-2,6-biphosphatase 3 (PFKFB3) gene is over-expressed in immune cells and increases concomitantly with glucose transporter-1 (GLUT-1), hexokinase-II, and proliferating cell nuclear antigen upregulation, demonstrating that induction of T-cell proliferation by mitotic agents is required for metabolic reprogramming. PI3K/Akt pathway inhibitors (e.g., Akti-1/2 and LY294002) can reduce PFKFB3 gene induction by phytohemagglutinin, as well as fructose-2,6-bisphosphate and lactate production [13]. Thus, the substances that affect the activation of PI3K/Akt signaling pathway may affect the glycolysis of T cells. For example, Acylglycerol kinase, as a specific lipid kinase, can induce the phosphorylation of PTEN, thereby inactivating PTEN and maintaining metabolism and function of CD8^+^ T cells [14]. Additionally, mTOR signaling pathway also regulates Treg glucose metabolism. A study has found that TCR activation promotes the assembly and activation of the mTORC1 complex of Treg on lysosomal membrane, while TRAF3IP3, a transmembrane molecule located on lysosomal membrane, can inhibit mTORC1 activity and its mediated glycolysis level. Thus, TRAF3IP3 maintains the stability and function of Treg [9]. Besides, epigenetic regulation is also important for the activation of signaling pathways. Just as deubiquitinating enzyme Otub1 can regulates T cell activation via inhibiting the activation of ubiquitin-dependent Akt [15].

In addition to the PI3K/Akt/mTOR signaling pathway, the transcription factor C-MYC-related pathway and nuclear receptor family pathway play important roles in glucose metabolism in T cells. C-MYC can enhance glycolysis by up-regulating the expression of GLUT-1 in activated T cells. As the key factor regulating the metabolic pathway to adapt to the requirements of T cells during activation, T cells without C-MYC cannot survive and differentiate [16].

Recent studies have also focused on nutrient transporters and enzymes related to glucose metabolism. For example, miR-143 regulates T cell differentiation by inhibiting GLUT-1 [17]. Autophagy related gene Atg5 can regulate the change of histone methylation, inhibit the metabolism and upregulation of the transcription of effector target genes (such as GLUT-1), thus inhibiting the glucose metabolism of CD8^+^ T cells and interferon (IFN)-γ secretion [18]. In studies on glycometabolism-related kinases, it was found that damage to glycolyze-related enzymes could inhibit CD8^+^ T cell function, such as ENOLASE 1 [19]. Acetate, a metabolite, enhances IFN-γproduction in exhausted T cells with an acetyl-CoA synthetase-dependent manner under low-glucose conditions [20].

Furthermore, memory T cells in pleural effusion secondary to lung cancer cannot upregulate CD71 and GLUT-1 when activated under hypoxic conditions, and the glycolysis is defective [21]. Hence, the metabolic changes of T cells in special cases are worthy of attention.

### Lipid metabolism

Lipid metabolism mainly includes fatty acid metabolism and cholesterol metabolism. Under hypoxic conditions, hypoxia-inducible factor (HIF)-1α induces pyruvate to leave mitochondria with OXPHOS, making Tregs dependent on fatty acids for mitochondrial metabolism in hypoxic tumors. Thus, FAO is crucial for Treg metabolism in cancer [22]. Besides, Teffs can obtain fatty acids for the microenvironment, while memory T cells only use carbon derived from glucose metabolism to synthesize fatty acids [23]. And lipid metabolism is also important for maintaining the balance between Teffs and Tregs [24].

Fatty acid synthesis (FAS) is mainly used to produce key lipid cell structures such as cell membrane necessary for cell proliferation, while FAO mainly provides ATP for cells and produces many metabolic intermediates with important physiological functions. During the synthesis of fatty acids, sterol regulatory element-binding protein (SERBP)-1 is activated by PI3K/Akt signaling pathway, and then ATP citrate lyase (ACLY) and fatty acid synthase (FASN) are up-regulated to promote the synthesis of fatty acids [25]. Activated T cells mainly rely on FAS [26], while naïve T cells and memory T cells maintain basic functions such as membrane functional integrity by increasing FAO [8]. FAO can inhibit the activation of Teffs by increasing programmed cell death protein 1 (PD-1) expression, promoting carnitine palmitoyltransferase 1A, one of the rate-limiting enzymes of FAO, and inhibiting IFN-γ secretion. While FAO can promote Treg cells generation through MAPK signaling pathway activation [27]. Besides, Tregs are important for immune homeostasis. By promoting SERBP-1-dependent lipid metabolism, Treg cells inhibit CD8^+^ T cells to produce IFN-γ, maintain the immunosuppression of tumor-related macrophages, and coordinate the tumor-related immunosuppression microenvironment [28]. Peroxisome proliferator-activated receptors (PPARs) can also regulate lipid metabolism, and its high activation is associated with immunosuppression. Increased PPAR-γ activity can inhibit lipolysis, limit T cells OXPHOS, and promote the differentiation of Tregs [29].

Cholesterol can participate in maintaining cell membrane homeostasis and is a synthetic raw material for vitamin D, bile acids, and steroid hormones. In-depth studies showed that the metabolism of immune cells in the tumor microenvironment was affected by the changes of the cholesterol. When T cells are activated, TCR activation promotes cholesterol synthesis by affecting the transcription of key enzymes in the cholesterol biosynthesis pathway (CBP). Meanwhile, liver X receptor (LXR) [30], SERBP-2, and acyl-CoA acyltransferase (ACAT)-1 play key regulatory roles in maintaining intracellular cholesterol stability [31, 32]. Early studies showed that the cholesterol levels of both whole cells and plasma membrane were markedly increased in activated CD8^+^ T cells. When T cells are activated, lipid mediators and cytokines promote T-cell migration, proliferation, and differentiation [11, 33–36]. ACAT-1 and ACAT-2 are two key genes encoding cholesterol esterification enzymes that convert free cholesterol to cholesteryl esters for storage. ACAT-1 was mainly expressed in CD8^+^ T cells. Upon CD8^+^ T cell activation, ACAT-1 was up-regulated in an early stage. Additionally, ACAT-1 deficiency inhibits cholesterol esterification but promotes biosynthesis of cholesterol, which can upregulate the cholesterol level in the plasma membrane of CD8^+^ T cells, eventually enhancing TCR clustering and signaling as well as the resulting in the more efficient formation of the immunological synapse [37]. However, recent studies found that cholesterol or its derivatives, through LXR Sumoylation, can reduce the binding of P65 to the IL-9 promoter and further inhibit the expression of IL-9, thereby inhibiting Tc9 cells differentiation and its anti-cancer response [38]. Given the contradictions in the role of intracellular cholesterol, the researchers found that high cholesterol in tumor-infiltrating lymphocytes (TILs) upregulate the expression of XBP1, an endoplasmic reticulum stress receptor, which further promotes the expression of the immune checkpoint and inhibit T cell function [39]. TILs, unlike CD8^+^ T cells cultured in vitro, are generally limited in their anti-tumor activity due to their expression of many inhibitory receptors [40]. Thus, studies on the effect of intracellular cholesterol on T cell function need to focus on the T cell types.

### Amino acid metabolism

In addition to glucose and lipid, cell growth and function are also dependent on amino acids. Apart from nucleotides and protein synthesis, amino acids participate in a variety of metabolic pathways.

The influx of branched chain amino acids (such as leucine and glutamine) is critical for Teff cell differentiation and function through mTORC1 activation. Glutamine or leucine expression levels can affect the activation and function of T cells. For example, down-regulation of glutamine and leucine metabolism has been shown to inhibit the differentiation of TH1 and TH17 effector T cells while maintaining Treg differentiation. When T cells are activated, key amino acid transporters can be up-regulated through the activation of metabolic regulators such as C-MYC. Additionally, glutamine is converted to glutamate by glutaminase. Decreased glutamine and leucine metabolism will reduce mTORC1 activity and C-MYC expression, resulting in blocked T cell activation [41]. This reduction in glutamate metabolism causes immune cells to develop into Treg cells. Meanwhile, glutaminase can enhance IL-2-mediated mTORC1 signaling pathway activation to promote TH17 differentiation and inhibit TH1 as well as cytotoxic lymphocyte (CTL) differentiation [42].

Similar to the secondary pleural effusion of lung cancer, ovarian malignant ascites can downregulate the GLUT-1 expression levels on CD4^+^ T cells, which leads to the defection of *N*-linked protein glycosylation, thereby promoting IRE1α-XBP1 activation. The activation of XBP1 regulates the glutamine transporters expression and further restrict the inflow of glutamine under glucose deprivation conditions, thereby inhibiting T cell infiltration and IFN-γ secretion [43].

## Effects of tumor metabolism on T cells

Tumor mainly use glycolysis to produce energy, which causes the microenvironment to become acidic and hypoxic; some metabolic intermediates can impair the anti-tumor effect of Teffs. Glucose competition, amino acid competition, oxygen competition, and lactic acid secretion in the tumor microenvironment promote formation of the immunosuppressive phenotype. Therefore, determining the influence of the tumor microenvironment on T cell metabolism will be helpful for developing methods to enhance the anti-tumor effect of T cells while also killing cancer cells.

### Tumor metabolism reprogramming indirectly regulates tumor microenvironment

Tumor cells have infinite proliferation potential, in contrast to normal cells. To meet the demand for unlimited proliferation, tumor cells alter their metabolic patterns in glucose metabolism. An increasing number of studies has shown that tumors are not a homogeneous mass of malignant cells, but rather a complex structure containing vascular and stromal cells that support the tumor as well as a diverse array of infiltrating immune cells including lymphocytes and myeloid-derived cells. These cells alter their metabolic mode to proliferate in their specific environment. This adaptation which involves energy metabolism changes in the tumor is known as metabolic reprogramming. Normally, tumor cells mainly use glycolysis to provide ATP for rapid growth and use glutamine, lipids, and other substances to promote proliferation [44, 45]. Tumor cells also consume large amounts of oxygen and amino acids in the process of metabolism. During tumor progression, changes in some metabolites mediate the changes in immunomodulatory molecules, which are important factors leading to immune escape. Additionally, tumor cells compete with immune cells in the microenvironment for the components required for their own metabolism, further inhibiting immune cell functions.

Therefore, tumor metabolic reprogramming promotes tumor cell growth, with the resulting metabolites indirectly regulating the tumor microenvironment and ensuring tumor progression.

### Lactic acid in the tumor environment affects T cell function

Warburg glycolysis allows cancer cells to consume glucose and increase lactic acid, glutamine, and CO_2_ production, resulting in acidification of the tumor microenvironment. The consumption of these metabolic substrates also negatively affects the high-metabolism of T cells. Lactic acid and other metabolites produced through these metabolic processes also inhibit the proliferation and function of T cells to varying degrees [46]. Lactic acid, as a glycolysis product, can inhibit the PI3K/Akt/mTOR pathway and thus inhibit T cell glycolysis [47, 48]. Additionally, acidification of the tumor microenvironment impairs Teffs to a much greater extent compared to in Tregs, mainly because Teffs acquire energy mainly through glycolysis, while Tregs can rely on fatty acid oxidation. Lactic acid and an acidic tumor microenvironment can promote high levels of cytokines secretion, monocarboxylate transporter 1 inhibition, and thus promote neovascularization as well as glycolytic flux decreation[49, 50]. Meanwhile, the decrease of pH in the tumor microenvironment not only increases the infiltration of CD8^+^ T cells and NK cells but also reduces the number of Tregs to maintain the immunosuppressive tumor microenvironment [51].

### Hypoxia in the tumor environment affect T cell function

Because tumor aerobic glycolysis consumes a large amount of oxygen, the tumor microenvironment is often anoxic. A study conducted at the Weizmann Institute of Science found that hypoxic cultured T cells killed tumor cells by releasing high levels of granzyme B, a destructive enzyme, but not perforin. Adoptive treatment with hypoxic T cells increases the survival time of mice with OVA-expressing B16 melanoma cells [52].

HIF-1α is the main transcriptional regulator in the cellular response to hypoxia and is the downstream target of GLUT-1, which facilitates glucose uptake. Under hypoxic conditions, high HIF-1α expression leads to loss of T cell anti-tumor function. When *HIF-1α* is knocked out, increased fatty acid catabolism improves peroxisome proliferator-activated receptor α signaling in CD8+ tumor-infiltrating lymphocytes [33]. Besides, HIF-1α inhibit the immunosuppressive function of Tregs, which causes the function of Tregs mainly dependent on free fatty acids in tumor microenvironment [22].

Moreover, other immune cells also affect the function of T cells in hypoxic microenvironment. For example, B cells can promote Tregs recruitment and CD8^+^ T cells exhaustion by secreting chemokines. Myeloid derived suppressor cells inhibit the metabolism of T cells by accumulating key amino acids, inhibit the activation of T cells by increasing PD-L1 expression, and regulate the homing of T cells by cleaving L-selectin. M2-type macrophages promote T cell nonreactivity by increasing NO and decreasing arginine production [53].

### Low glycose in the tumor environment affects T cell function

Hypoxia and low glycose may send out opposite metabolic signals for T cells. T cells in the tumor microenvironment undergo glucose deprivation, leading to activated T cell hypo-responsiveness [45]. In T lymphocytes, glucose uptake and catabolism are not simply metabolic processes for nutrient utilization and energy generation. Glycolysis plays a key role in T cell differentiation from naïve T cells into tumor antigen-specific T effectors [5, 54]. Thus, by creating a microenvironment condition of glucose starvation for T cells, cancer inhibits the differentiation and expansion of tumor-specific T cells exposed to tumor-associated antigens, rendering them unable to develop into tumor-specific T effectors. Additionally, a low-glucose microenvironment can reduce the glycolysis function of T cells by reducing AKT activity and induce apoptosis of tumor-infiltrating T cells by activating the pro-apoptotic protein family [55, 56]. These metabolic conditions also promote T cells differentiation into Tregs. Besides, CD8^+^ TILs increased FAO in the presence of both hypoglycemia and hypoxia [33]. Furthermore, oxidative neutrophils also inhibit T cell function under hypoglycemia [57]. Therefore, the regulation of T cell function requires the consideration of various metabolic factors.

### Metabolic intermediates in the tumor environment affect T cell function

Metabolic intermediates produced by tumors such as tryptophan, kynurenine, and other molecules can also promote Treg differentiation and immunosuppressive function. Indo-leamine 2,3-dioxygenase (IDO) expression in tumor cells is related to tumor progression [58] and is an enzyme that degrades tryptophan [59]. Upregulation of IDO activity reduces tryptophan infiltration and induces T cell apoptosis. Tumor cells must compete for energy needed for growth while diminishing Teff anti-tumor responses [8]. The lipid metabolite prostaglandin E2 (PE2) is a class of highly active inflammatory mediators that promote tumor cell survival, proliferation, invasion, metastasis, and angiogenesis. Recent studies have shown that PE2 secreted by tumor cells can stimulate the secretion of cancer-promoting CXCL1, interleukin-6, and granulocyte colony-stimulating factor by myeloid cells and inhibit tumor necrosis factor-α secretion by lipopolysaccharide-stimulated myeloid cells [60].

## Treatments targeting T cell metabolism

T cells undergo metabolic reprogramming during proliferation, differentiation, and execution of effector functions. Some key signal pathways involved in metabolic reprogramming can change the energetic status. Metabolic competition in the tumor microenvironment is a new mechanism leading to strong inhibition of T cells. Therefore, it will be a new challenge for studies of anti-tumor immunotherapy to find a way are needed to develop methods for destroying the metabolism of tumor cells and while improving the ability of immune cells to obtain nutrients.

### Targeting T cell glucose metabolism

PD-1 ligand (PD-L1) expression by tumor cells activates the AKT/mTOR pathway to promote tumor cell glycolysis. Antibodies that block the PD-1/PD-L1 checkpoint may restore glucose levels in the tumor microenvironment, permitting T cell glycolysis and IFN-γ production [61]. PD-1, which is constitutively highly expressed, is considered as a surface marker of depleted CD8^+^ T cells [62]. T cells with PD-1 activation are unable to utilize glucose and branched chain amino acids, but the ratio of FAO is increased [7]. Hypoxia stimulates the expression of PD-L1 on tumor cells to suppress the T-cell killing tumor ability [63]. Thus, PD-1/PD-L1 inhibitors can help T cells kill tumors by regulating T cell metabolism.

Similar to PD-1, lymphocyte activation gene (LAG)-3 is also an inhibitory molecule on T cells. It prevents excessive proliferation of naïve T cells by inhibiting IL-7-mediated STAT5 activation. Due to increased mitochondrial content, LAG-3-deficient naïve T cells showed increased oxidation and glycolytic metabolism. So, targeting LAG-3 is expected to provide new ideas for anti-tumor therapy by regulating the metabolism of T cells [64].

Recent studies also showed that monoclonal antibodies blocking cytotoxic lymphocyte antigen 4 (CTLA-4) have been widely used in both hematologic and solid tumors [65]. CTLA-4 is constitutively expressed on the surface of chronic activated T cells. CD28 and CTLA4 share identical ligands: CD80 and CD86. Due to CTLA4 has a much higher affinity for both ligands, it can inhibit T cell activation by outcompeting CD28 in antigen-presenting cells such as dendritic cell and delivering inhibitory signals to cells [66]. Activated CTLA-4 competes with B7 ligand and recruits protein phosphatase 2, a phosphatase that regulates the cell cycle, which inhibits the PI3K/Akt/mTOR signaling pathway and inhibits glucose uptake, thereby inhibiting T cell metabolism and proliferation [8].

Imatinib, a BCR-ABL kinase inhibitor, has shown opposing effects on T cell metabolism. It can activate CD8^+^ T cells and induce Treg cell apoptosis by down-regulating IDO expression [65]. Imatinib can also decrease glucose uptake from the media by switching from glycolysis to mitochondrial glucose metabolism in BCR-ABL-positive cells [67].

As a classical regulator of glucose metabolism, metformin has direct antitumor activity and indirect CTL killing effect. By activating the LKB1-AMPK system, the mTOR pathway can be inhibited to interfere with glycolysis in the tumor, thereby inhibiting tumor growth. Besides, the regulation of metformin on PD-L1 is related to the glycosylation of PD-L1, which further promotes the activation of endoplasmic reticulum related protein degradation pathway, resulting in the downregulation of PD-L1 expression and enhancing the killing effect of CTL [68].

PIM kinase is an immune regulatory kinase that also participates in T cell glucose metabolism. mTORC1 activity can be enhanced by inhibiting PIM kinase, which improves the uptake of glucose by T cells and increases the anti-tumor function of T cells [69].

In addition to molecule targets, the products of some metabolic processes can also serve as therapeutic targets. For example, phosphoenolpyruvate (PEP) is an intermediate metabolite produced by glucose metabolism. The enzyme phosphoenolpyruvate carboxykinase-1 is overexpressed during PEP production in CD4^+^ and CD8^+^ T cells, which can significantly upregulate the effector function of T cells and inhibit tumor growth, prolonging the survival of melanoma-bearing mice [45]. Due to the tumor microenvironment, the specific accumulation effect of some nanoparticle-mediated therapeutics can be used to improve the anti-tumor efficacy. For instance, the combination of oxaliplatin prodrug and PEGylated photosensitizer into a single nanoplatform can promote T cell antitumor immune response by immunogenic cell death [70]. By knocking down LDHA by RNAi nanoparticles, pyruvate metabolism is reprogrammed to reduce lactic acid production [51].

### Targeting T cell lipid metabolism

Recent studies showed that metabolic reprogramming occurs in tumor cells and immune cells, intracellular cholesterol levels are significantly up-regulated in cancer cells, and their metabolites are abnormally accumulated during the development of tumor cells. However, the effect of traditional lipid metabolism drugs on T cells remains controversial, such as statins. It was found to have anti-tumor functions by inhibiting lipid metabolism in tumors and reducing the cholesterol level of T cells, thereby inhibiting the function of CTL cells [71]. On the other hand, it may down-regulates T cell expression of PD-1, 2B4, TIM-3, and LAG-3 [39].

The mevalonate kinase (MVK) metabolic pathway is involved in cholesterol synthesis. Blocking the rate-limiting enzyme of the MVK pathway in tumor cells can significantly reduce downstream metabolic production of the MVK pathway. Some studies showed that tumor cells that continuously express high MVK metabolic pathway levels can activate the immune response, revealing a new anti-tumor target for tumor immunotherapy. MVK is also crucial for T cell activation in an AKT/mTOR signaling-dependent manner [72].

Furthermore, the ACAT-1 inhibitor avasimibe not only inhibits cholesterol esterification in tumor cells, but also increases the intracellular free cholesterol level, thereby inhibiting the proliferation and metastasis of tumor cells and enhancing the activity of CD8^+^ T cells. Avasimibe has also been used to treat cancer in tumor-model mice and showed good anti-tumor effects. A combination of avasimibe and a PD-1 antibody showed better efficacy than monotherapy in controlling tumor progression [37].

In the microenvironment with hypoglycemia and hypoxia, most of the T cells were inactivated with inhibitory receptors (such as PD-1 and LAG-3) up-regulation, and the free fatty acids around them were significantly increased. Fenofibrate can increase the FAO of T cells by activating PPAR-α, thus reversing the inhibitory effect of T cells in the microenvironment [33].

### Targeting T cell amino acid metabolism

Indoleamine-2,3-dioxygenase 1 (IDO1) catalyzes the oxidation of tryptophan into kynurenine and is partially responsible for acquired immune tolerance associated with cancer. Some studies showed that IDO expression is associated with low T cell infiltration and reduced survival in colorectal cancer [73]. Additionally, IDO induces Treg cell generation via an aryl hydrocarbon receptor-dependent mechanism [68].

The IDO1 small molecule inhibitor navoximod (GDC-0919) is active as a combination therapy in multiple tumor models and relieves CD8+ T cell inhibition by degrading tryptophan [74]. One novel IDO inhibitor, INCB024360, showed effectiveness in mouse models by increasing T cell proliferation and IFN-γ production [61]. Thus, the development of IDO inhibitors is one of the T cell activation modalities currently being explored.

Studies of adoptive immunotherapy showed that PD-1 expression was decreased in CD8^+^ T cells cultured under glutamine-limited conditions, while Ki67 and pro-survival factor expression was increased. Therefore, a novel approach for culturing CD8^+^ T cells under glutamine restriction may be a promising strategy for improving adoptive immunotherapy [75]. Similarly, *N*-acetylcysteine can inhibit FOXO1 expression by activating the PI3K/AKT signaling pathway, thus affecting granzyme B secretion and PD-1 expression to further increase the anti-tumor ability of T cells amplified in vitro [76].

## Conclusion

**Fig. 2:**
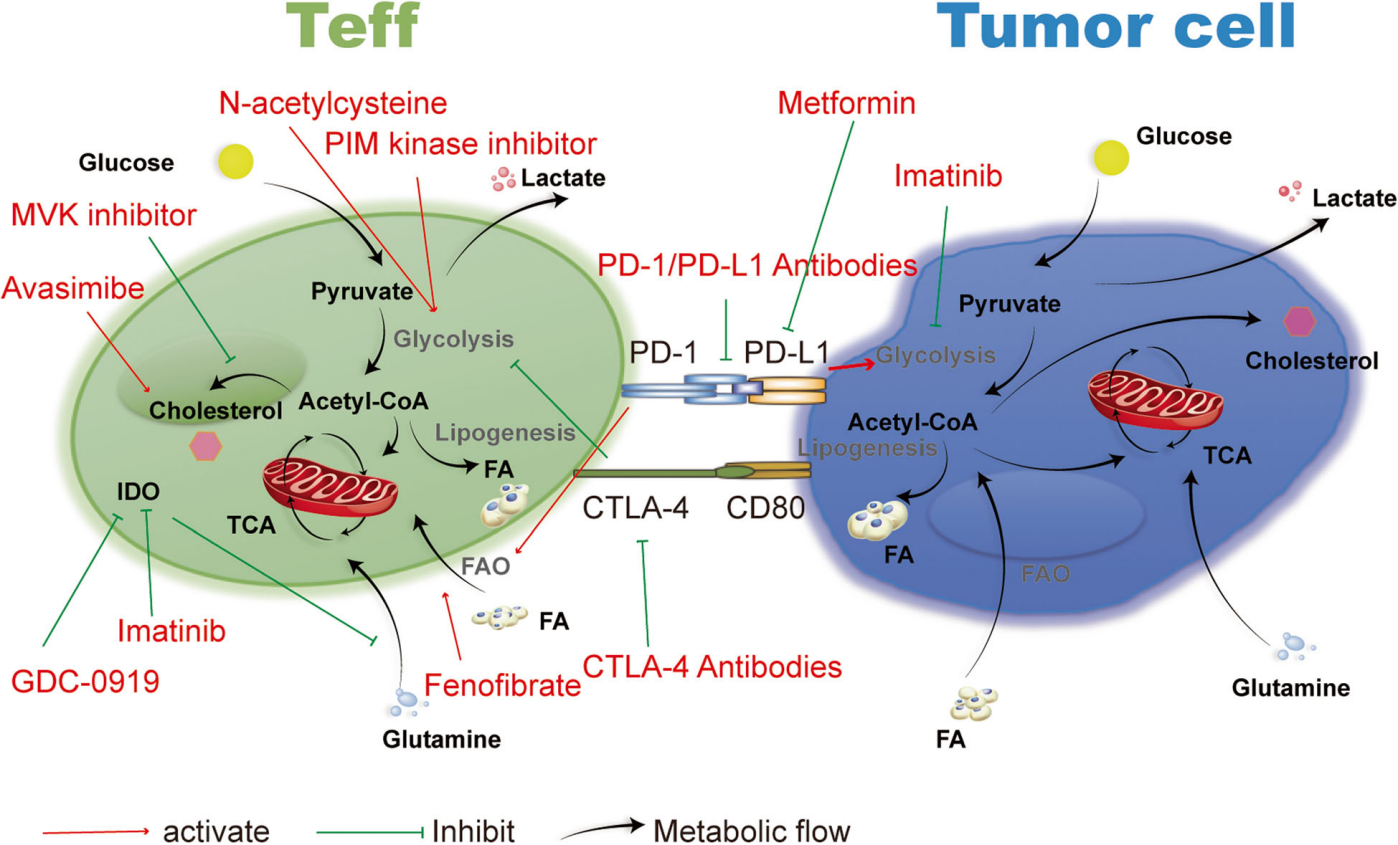
Therapeutic targets and drugs against tumor metabolism. Tumor cells compete with immune cells in the microenvironment for the components required for their own metabolism, further inhibiting immune cell functions. Some drugs which focus on the metabolic processes of T cells and tumor cells contribute to the anti-tumor effect, such as PD-1/PD-L1 antibodies, metformin, and imatinib

T cell metabolism can be altered to perform different cellular functions. To fulfill the rapid growth and produce energy, metabolism in T cells is switched from OXPHOS to glycolysis and glutamine metabolism to support cell growth and proliferation as well as lipid and nucleotide synthesis. Therefore, determining the reasons for T cell differentiation in the tumor microenvironment is helpful for clarifying the metabolic requirements and regulatory modes of different T cell subtypes. Metabolic reactions in tumor cells and immune cells are regulated by nutrients and metabolites in the microenvironment. By studying glucose, amino acid, and lipid metabolism pathways in tumor and T cells, novel anti-tumor therapeutic targets may be revealed. (Fig. 2, Table 1) However, balancing the inhibition of tumors and maintenance of immune cell activity remains challenging. In addition to the roles of checkpoint inhibitors, which directly affect tumor cells, the metabolism of immune cells requires further analysis. To evaluate specific metabolic pathways, metabolites and metabolic enzymes that regulate T cell metabolism to enhance the ability of T cells to kill tumors and exert anti-tumor effects on tumor cell metabolism require additional analysis. Due to the competition of nutrients between tumor and T cells, the metabolic adaptation of cells to microenvironment is the key to maintain the cell function. Besides, since the interaction between immune cells can affect the tumor suppressive microenvironment, future research may focus on the mechanism of same metabolic molecule in different cells. Above all, the discovery of drugs that can both enhance anti-tumor immunity and directly kill tumors, such as imatinib and ACAT-1 inhibitors, is the focus of future drug development.

**Table 1:** Metabolism targeting approaches of T cell and tumor cells

Name	Target	Signaling pathway	Effect
PD-1/PD-L1 Anitibodies	PD-1/PD-L1	PI3K/Akt/mTOR	Teffs: increase FAO
			Tumor: inhibit glycolysis
CTLA-4 Antibodies	CTLA-4	PI3K/Akt/mTOR	Teffs: inhibit glucose uptake
Imatinib	BCR-ABL kinase/IDO	BCR/ABL IDO	Teffs: activate
			Treg: apoptosis
			Tumor: switching from glycolysis to OXPHOS
Metformin	PD-L1	LKB1-AMPK system mTOR	Tumor: down-regulate PD-L1 expression
PIM kinase inhibitor	PIM kinase	mTORC1	Teffs: increase glucose uptake
Enzyme phosphoenolpyruvate carboxykinase-1	Phosphoenolpyruvate	Sarco/ER Ca(2+)-ATPase (SERCA) activity	Teffs: upregulate the effector function
MVK inhibitor	MVK	PI3K/Akt/mTOR	Teffs: promote activation
			Tumor: inhibit
Avasimibe	ACAT-1	Cholesterol esterification	Teffs: activate
			Tumor: inhibit the proliferation and metastasis
GDC-0919	IDO1	tryptophan	Teffs: relieves CD8+ T cell inhibition
INCB024360	IDO	tryptophan	Teffs: increase proliferation and IFN-γ production
*N*-acetylcysteine	FOXO1	PI3K/Akt/mTOR	Teffs: affect granzyme B secretion and PD-1 expression

## Data Availability

Not applicable

## Abbreviations

ACAT: Acyl-CoA acyltransferase
ACLY: ATP citrate lyase
CBP: Cholesterol biosynthesis pathway
CTL: Cytotoxic lymphocyte
CTLA-4: Cytotoxic lymphocyte antigen 4
FAO: Fatty acid oxidation
FAS: Fatty acid synthesis
FASN: Fatty acid synthase
IDO: Indoleamine 2,3-dioxygenase
LXR: Liver X receptor
MVK: Mevalonate
OXPHOS: Oxidative phosphorylation
PD-1: Death protein 1
PD-L1: Death protein 1 ligand
PEP: Phosphoenolpyruvate
PPAR: Peroxisome proliferator-activated receptors
SERBP: Sterol regulatory element-binding protein
TCR: T-cell antigen receptor
Teff: Effector T cell
TILs: Tumor infiltrating lymphocytes
Tregs: Regulatory T cells
